# Effects of Three Different Withering Treatments on the Aroma of White Tea

**DOI:** 10.3390/foods11162502

**Published:** 2022-08-19

**Authors:** Huiting Wu, Yuyu Chen, Wanzhen Feng, Shanshan Shen, Yuming Wei, Huiyan Jia, Yujie Wang, Weiwei Deng, Jingming Ning

**Affiliations:** State Key Laboratory of Tea Plant Biology and Utilization, Anhui Agricultural University, Hefei 230036, China

**Keywords:** white tea, withering treatment, volatile compounds, aroma extract dilution analysis, odor activity value

## Abstract

White tea (WT) is a slightly fermented tea, and withering is a critical step in its processing. The withering treatment can affect white tea’s aroma; different treatments’ effects were investigated in this study. White tea was withered indoors (IWT), in a withering-tank (WWT), or under sunlight (SWT). Quantitative descriptive analysis (QDA) results showed that SWT had a more obvious flower aroma, and WWT had a more pronounced grassy aroma. Volatile compounds were extracted and subsequently detected with solvent-assisted flavor evaporation (SAFE) and headspace solid-phase microextraction (HS-SPME) combined in addition to gas chromatography–mass spectrometry (GC-MS). A total of 202 volatile compounds were detected; 35 of these aroma-active compounds met flavor dilution (FD) factor ≥ 4 or odor activity value (OAV) ≥ 1. The nine key potent odorants for which both conditions were met were *dimethyl sulfide*, *2-methyl-butanal*, *1-penten-3-one*, *hexanal*, *(Z)-4-heptenal*, *β-Myrcene*, linalool, geraniol, and trans-β-ionone. These results were used with QDA to reveal that SWT had a stronger floral aroma mainly due to an increase of geraniol and linalool. Moreover, WWT had a stronger grassy aroma mainly due to increased *hexanal*. The results could be used to select processing methods for producing white tea with a superior aroma.

## 1. Introduction

White tea is primarily produced in Fujian Province, China, and is one of six major tea types consumed in China [1]. The flavor of white tea is the result of its unique processing. White tea is naturally withered and dried but is not fixed or rolled [2]. Withering is a key step that determines the quality of white tea by causing a series of changes in the internal composition of the tea leaves due to the gradual loss of water and the activation of various enzymes under certain external temperature and humidity conditions [3]. Various withering methods are used, including withering indoors, in a withering-tank, and under sunlight [4]. In indoor withering, the fresh leaves are placed on a bamboo plaque and allowed to wither naturally. In the withering-tank method, fresh leaves are laid thickly in a tank and heated with a blower. Finally, in sunlight withering, fresh leaves are laid on a bamboo plaque and placed outside in sunlight during the spring [5]. For black tea, red light withering treatments are used in the summer to improve the aroma of the tea significantly [6]. Moreover, short-term sunlight withering treatment can increase aromatic compounds in oolong tea [7]. Thus, specific withering treatments produce differences in the tea aroma.

White tea is prized for its fresh and elegant aroma. Hexanal, linalool, (E)-2-hexenal, phenylethyl alcohol, benzaldehyde, and methyl salicylate are the primary volatile compounds that determine the aroma of white tea [8]. The increase of free amino acids and the decrease of glycoside-bound volatile content during the withering process promotes the formation of the tea’s unique aroma [9]. Increasing the withering time and the thickness of the spread leaves or using light-emitting diode light irradiation can improve the freshness and purity of the white tea aroma [10,11]. Although numerous studies have investigated withering treatment methods, the effects of different withering treatments on aroma have not yet been studied and remain unclear.

Most investigations of white tea aroma use headspace solid-phase microextraction (HS-SPME) to extract and analyze volatile compounds. One such analysis that employed HS-SPME revealed that the aromas of four white teas of different tenderness exhibited significant differences in aroma [12]. A comparison of naturally aged white teas by using HS-SPME revealed that the rapid aging technique could enhance floral and herbal aromas [13]. However, HS-SPME is ineffective for extracting weakly volatile compounds [14]. Solvent-assisted flavor evaporation (SAFE) is a method that enables the isolation of volatile compounds from complex matrices in a low-temperature vacuum environment [15]. The volatile compounds extracted using SPME and SAFE were found to be mutually complementary in dried fermented sausages [16]. HS-SPME is effective for capturing intensely volatile compounds, whereas SAFE has poor performance for extracting these compounds because they tend to dissipate during the concentration process [14]. Combining HS-SPME with SAFE enables a more comprehensive analysis of the differences in volatile compounds between teas. To the best of our knowledge, no relevant studies have investigated the extraction of volatile compounds from white tea using HS-SPME and SAFE.

The molecular sensory science approach is a powerful tool for choosing key odor-active compounds and revealing the reasons for differences in aromas between teas [17,18]. In this study, white teas were collected by withered indoors (IWT), in a withering-tank (WWT), or under sunlight (SWT), and their volatile compounds were comprehensively extracted using SAFE and HS-SPME. The analysis was performed by combining a molecular sensory science approach with chemometric methods to compare the effects of the three withering treatments on the white tea aroma and to elucidate the reasons for any differences.

## 2. Materials and Methods

### 2.1. Chemicals and Samples of Different Withering White Teas

Distilled water was obtained from Watsons Water Company (Guangzhou, China). Dichloromethane was purchased from TEDIA (Fairfield, OH, USA) and was chromatographically pure; it was used for experiments after redistillation. Ethyl decanoate was 99% pure and was purchased from Aladdin (Shanghai, China) and was diluted to 1 mg/mL in absolute ethanol as an internal standard. Finally, n-alkanes (C6–C40; Sigma-Aldrich, St. Louis, MO, USA) were used to calculate retention indices to identify volatile compounds.

**Figure 1:**
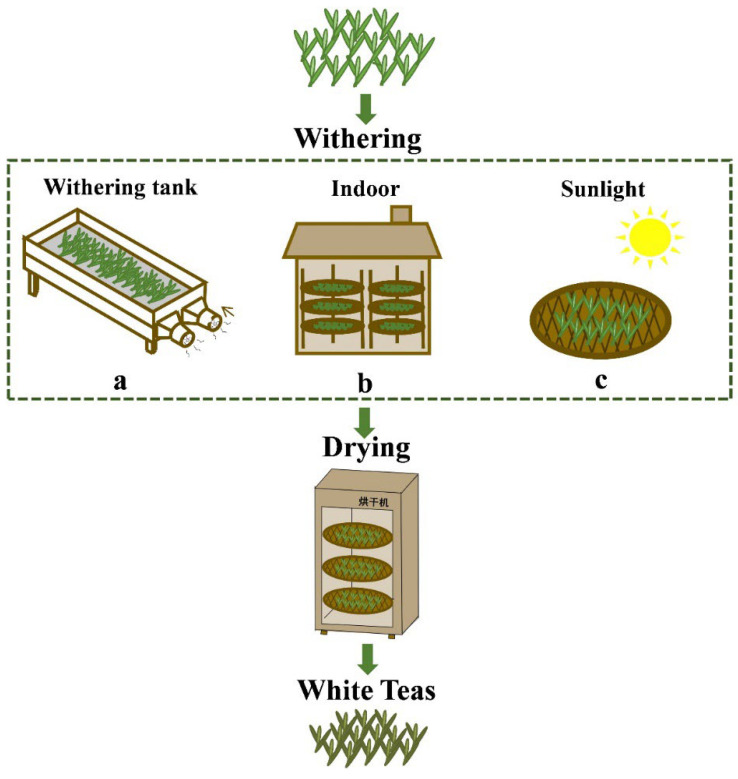
White tea processing flowchart. (**a**) Withering-tank treatment, (**b**) indoor treatment, and (**c**) sunlight treatment.

The samples were produced at the Zhongcha factory (Songxi County, Nanping City, Fujian Province, China). Fresh leaves (*Camellia sinensis* (L.) O. Zhenghe Dabai) with one bud and two or three leaves were picked in April 2021. Two local tea masters with more than 20 years of experience in white tea production monitored the tea leaves in turn. The fresh tea leaves were evenly distributed for withering in accordance with traditional processing methods in the three aforementioned approaches. (Figure 1). The number of fresh leaves used in each method is adjusted according to the capacity of the withering tank and the bamboo plaque, with approximately 50 kg of fresh leaves used in the withering tank and 10 kg of fresh leaves used in each sunlight withering and indoor withering. For SWT, the fresh leaves were spread on a bamboo plaque for withering under the sunlight during the day; the plaques and tea were retrieved and placed indoors at night (Figure 1a). For IWT, fresh leaves were laid on bamboo plaques and placed in a room to wither (Figure 1b). For WWT, fresh leaves were placed in a withering-tank comprising a tank and an engine (YY802-4, Shanghai Guantao Electromechanical, Shanghai, China) and withered by blasting at 30 °C (Figure 1c). To ensure a consistent degree of withering among the samples, the moisture of the leaves at the end of withering was controlled at about 10%, as shown in Table S1. Moreover, to ensure the quality of white teas, the finished samples were placed in a tea dryer (6CHZ-9B, Fujian Tea Machinery Intelligent Technology, Fujian, China) at 85 °C until they were completely dry after the completion of withering was confirmed by the tea masters again.

Sunlight withering, withering tank withering, and indoor withering were performed once, and three different batches of samples were made for each treatment. After returning to the laboratory, 200 g of samples from different batches of the same treatment were mixed separately, packed in tinfoil bags, and stored in a −20 °C refrigerator. Finally, three finished white teas produced using SWT, IWT, and WWT were collected.

### 2.2. Quantitative Descriptive Analysis

Quantitative descriptive analysis [19] (QDA) was performed in an odorless room at 22 ± 1 °C to accurately describe and analyze the tea aroma characteristics. The sensory panel comprised 12 panelists: 6 men and 6 women aged 22 to 31 years who were current master’s students and faculty from the Tea Science laboratory at Anhui Agricultural University. All the panelists had passed a training course in “tea sensory evaluation” and systematically learned about typical aroma types and descriptive vocabulary of white tea. Prior to the start of the experiment, all the experimenters received a total of more than 40 h of sensory recognition training over four weeks. The experiment comprised two parts: first, panelists identified the aroma descriptors of the tea samples and selected the top six aroma descriptors by voting, namely grassy, sweet, fermented, fruity, floral, and soy milk; the selected aroma descriptors were then scored as follows: 0–2 (very weak), 2–4 (weak), 4–6 (moderate), 6–8 (strong), 8–10 (very strong), according to a previously reported method [20,21]. The sniffing was repeated three times for each person in each experiment.

### 2.3. Solvent Assisted Flavor Evaporation 

Three grams of tea were steeped in 150 mL of deionized water for 5 min. The tea leaves were then quickly filtered, and the tea infusion was placed in an ice water bath to cool rapidly to room temperature. A solution of ethyl decanoate was produced by diluting 1 mg of ethyl decanoate with 2 mL of absolute ethanol; the resulting solution was then diluted to 5 mg/L with deionized water. Subsequently, 1 mL of this 5 mg/L ethyl decanoate solution was added to the tea infusion as an internal standard. Each tea infusion was extracted three times in succession with 30 mL of dichloromethane, and the lower organic layer was collected and dried using anhydrous sodium sulfate at room temperature. This tea infusion was distilled under a vacuum of 10^−3^ Pa at 40 °C in accordance with the SAFE procedure [15]. The distilled tea infusion was then concentrated to 2 mL using the TurboVap evaporation system (Biotage, Shanghai, China), dried again with anhydrous sodium sulfate, and then concentrated to 100 μL under low airflow. This concentrated aroma was used for GC-MS analysis.

### 2.4. Headspace Solid-Phase Microextraction

Volatile compounds were extracted by HS-SPME using 50/30 μm polydimethylsiloxane/divinylbenzene/carboxen (PDMS/DVB/CAR) SPME fibers (Supelco, Bellefonte, PA, USA). The fibers were analyzed after pretreatment at 300 °C for 30 min at the GC inlet. The preparation of the tea infusion was identical to that of the SAFE method. A total of 10 mL of cooled tea infusion, 3 g NaCl, and 10 μL of 5 mg/L ethyl decanoate were placed in a 20 mL headspace bottle and immediately sealed. The bottle was then placed in a water bath at 30 °C for 15 min; the SPME fibers were subsequently passed through the pores to enable volatile adsorption at 1 cm above the liquid for 30 min. The fibers were then immediately inserted into the GC-MS injector for thermal desorption.

### 2.5. Gas Chromatography-Mass Spectrometry 

An Agilent 7890A-5975C MSD inert GC-MS system (Agilent, Santa Clara, CA, USA) equipped with an Agilent DB-5MS (30 m × 0.25 mm × 0.25 μm) capillary column was used. High-purity helium (99.999% purity) at 1 mL/min was used as the carrier gas.

For SAFE samples, the oven temperature was maintained at 40 °C for 5 min, increased to 90 °C at 10 °C/min and maintained for 2 min, then increased to 200 °C at 4 °C/min, and finally increased to 250 °C at 25 °C/min and maintained for 10 min. The injector temperature was then set to 250 °C (DB-5MS), and the sample was injected in splitless mode (1 μL). For HS-SPME samples, the oven temperature was increased from 40 °C to 100 °C at a rate of 3 °C/min, then to 130 °C at a rate of 2 °C/min, and finally to 250 °C at a rate of 40 °C/min. The other conditions were identical to those for the SAFE sample.

### 2.6. Gas Chromatography-Olfactometry and Aroma Extract Dilution Analysis

A stepwise dilution experiment was used to measure the relative contribution of individual odorants with reference to a previous related study [18]. GC-O experiments were performed by three team members. Aroma extract dilution analysis (AEDA) was employed to dilute the samples in equal concentration gradients.

For SAFE samples, dichloromethane was used to progressively dilute the samples at a ratio of 1:2 (1:2, 1:4, 1:8, …, 1:512). Each panelist sniffed the sample through the sniffer port and was asked to indicate the time at which they sniffed the odor, describe the odor, record the time, and report the relative odor intensity. The HS-SPME samples were used to identify highly volatile compounds. Odor intensity was assessed on a scale of 0–4, with 0–1 indicating weak, 1–2 indicating moderate, and 3–4 indicating strong. On the basis of the preliminary data and odor intensity, WWT was selected due to a higher score, and the GC-MS was set to separation mode (split ratios of 1:1, 1:3, 1:7, ..., 1:511) for the AEDA. The flavor dilution (FD) factor of each odorant was calculated as the maximum dilution ratio for which panelists reported smelling an aroma.

### 2.7. Calculation of Odor Activity Values

The odor activity value [22] (OAV) of a compound is defined as the ratio of its concentration in the tea infusion to its odor threshold in water. It can be calculated with the equation OAV = C/T, where C is the concentration and T is the odor threshold. These odor thresholds were obtained from the literature [12,23,24] and Kreissl J, Mall V, Steinhaus P, Steinhaus M. Leibniz-LSB@TUM Odorant Database, Version 1.2. [25] (Leibniz Institute for Food Systems Biology at the Technical University of Munich: Freising, Germany, 2022)

### 2.8. Compound Identification and Quantitative Analysis

Unknown compounds were identified by matching their retention indices, odor descriptions, and mass spectra to reference compounds in the NIST 17 library. In accordance with the method [26], the retention index (RI) on GC-MS was calculated for each volatile compound in each sample and was compared with the RI values obtained from the National Institute of Standards and NIST 17 library; compounds with an RI difference of >20 were deleted. Semi–quantification was performed according to the relative peak areas of volatiles to ethyl decanoate using a DB-5MS column. Deconvolution was performed using the AMDIS software (AMDIS, Version 2.72, NIST, Gaithersburg, MD, USA) to separate overlapping peaks, and the Chemstation software (Agilent) was employed for mass map matching and peak area calculations.

### 2.9. Statistical Analysis

All experiments were repeated three times, and data were expressed as the mean and standard deviation; one-way ANOVA using Duncan’s test was performed, and *p* < 0.05 indicates statistically significant differences. All statistical analyses were performed using SPSS Statistics software (Version 25, SPSS Inc., Chicago, IL, USA).

Principal Component Analysis (PCA) and Hierarchical Cluster Analysis (HCA) are mathematical tools that represent variation in a data set in terms of several factors and allow an unsupervised visual assessment of similarities and differences between samples [27,28]. PCA and HCA were performed with the SIMCA-P 14.1 software (Umetrics, Umea, Sweden). In these analyses, the quality of the models was evaluated using the relevant R^2^ and Q^2^ values.

## 3. Results and Discussion

### 3.1. Aroma Characteristics of White Teas Using Different Withering Treatments

**Figure 2:**
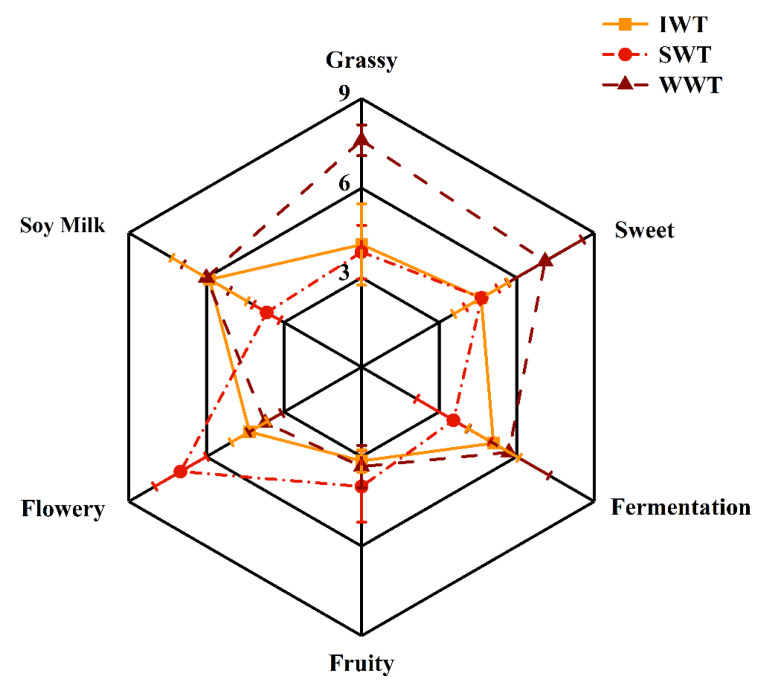
Radar plot for quantitative descriptive analysis (SWT: sunlight withering-treated white tea; WWT: withering-tank withering-treated white tea; IWT: indoor withering-treated white tea).

The radar plot in Figure 2 reveals that the three withering treatments result in white tea with different aroma characteristics. The results revealed that these three white teas scored similarly for fruity aroma. SWT scored the lowest for soy milk and fermented aromas but had the highest intensity scores for floral aroma. WWT had the highest scores for grassy and sweet aromas. Thus, the overall aroma characteristics differed between withering treatments; the clearest differences were the pronounced floral aroma of the SWT and the stronger herbal and sweet aromas of WWT. These disparities may be caused by specific compounds produced in white tea during the different withering treatments, resulting in changes in the concentration of some compounds and thus changes in the intensity of the aroma characteristics [14]. 

### 3.2. Comparison of the Differences in the Overall Volatile Compounds of White Teas

The three white teas detected a total of 202 compounds (Table S2). Most of the compounds were alcohols or aldehydes, which is consistent with the known aroma composition of white tea; these compounds are likely produced by the hydrolysis of glycosides, oxidative degradation of fatty acids, or carotenoids, or Maillard reactions during white tea processing [9].

**Figure 3:**
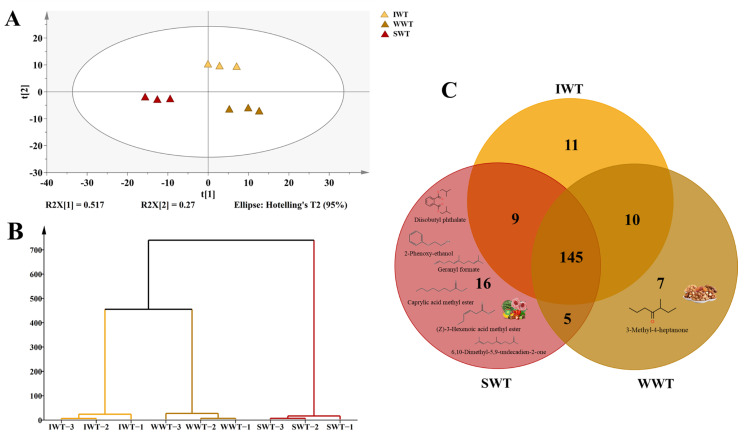
Overall differences in volatile compounds among white teas. (**A**) Principal component analysis, (**B**) hierarchical cluster analysis, (**C**) Venn diagram; diisobutyl phthalate, geranyl formate, caprylic acid methyl ester, (Z)-3-hexenoic acid methyl ester, 2-phenoxy-ethanol, 6,10-dimethyl-5,9-undecadien-2-one are volatile compounds with floral or fruity odor and are only present in SWT. 3-Methyl-4-heptanone are volatile compounds with nutty odor and are only present in WWT (SWT: sunlight withering-treated white tea; WWT: withering-tank withering-treated white tea; IWT: indoor withering-treated white tea).

The 202 volatile compounds extracted from the three samples were analyzed using PCA, and fractograms were obtained. The PCA results indicated that the first two principal components explained 51.7% and 27%, respectively, of the total variance (Figure 3A). According to the results, IWT, SWT, and WWT samples were clustered into different quadrants and well-separated, indicating that these three samples differ. The HCA results revealed that the samples were divided into three clusters; SWT samples were further divided into separate clusters, indicating greater differences among SWT samples (Figure 3B). This result is consistent with the PCA results.

The volatile compounds detected in the three white teas were analyzed, and Venn diagrams depicting commonalities between the teas were drawn (Figure 3C). The three white teas shared 145 volatile compounds. SWT shared five and nine volatile compounds with WWT and IWT, respectively. WWT and IWT shared ten volatile compounds. SWT, WWT, and IWT had 16, 11, and 10 unique volatile compounds, respectively. Thus, different withering treatments can indeed result in the production of different volatile compounds. This is consistent with the results of previous studies, which have reported that different tea leaf processing methods could result in different aromas [29].

GC-MS results cannot directly confirm the degree of contribution of compounds to the aroma of a sample because of the influence of certain component thresholds. Therefore, the OAVs and FD factors were used to determine the degree of contribution of each compound to the overall aroma [30]. Volatile compounds with FD factor ≥ 4 or OAV ≥ 1 were defined as odor-active compounds [18]. Most of the 16 volatile compounds present in the SWT only exhibited floral or fruity aromas; these compounds include geranyl formate (fresh rose), methyl benzoate (starfruit-like and sweet), octanoic acid, methyl ester (fruity), diisobutyl phthalate (mildly fragrant), 2-phenoxy-ethanol (mild rose aroma), and 6,10-dimethyl-5,9-undecadien-2-one (sweet and rose). These results are consistent with those of QDA, indicating that the high scores for floral characteristics in the SWT could be attributed to these volatile compounds; however, the specific contributions of these odor-active compounds require further investigation. A study demonstrated that black tea treated with red light during withering has a significantly stronger floral aroma than that treated with white light because of the effect of light on glycosidase activity [6]. Therefore, light may promote the activity of certain enzymes, resulting in increased production of corresponding volatile compounds and increasing the strength of the floral aroma of SWT. 3-Methyl-4-heptanone (nutty) is a volatile compound identified only in WWT. Table 1 indicates that this compound is odor active. Ketones are produced through the oxidation of lipids, degradation of amino acids, and the Maillard reaction [31]. The withering process was accelerated by hot air in the WWT; ketones with a nutty aroma may have been produced during this process as a result of these reactions. However, the QDA results did not indicate a significant effect on the tea aroma; this may be attributed to the interactions between compounds.

### 3.3. Comparison of the Differences in Key Odor-Active Compounds of White Teas

Table 1 indicates 20 identified odor-active compounds with OAV ≥ 1. *Dimethyl sulfide* (cabbage), *hexanal* (green, grassy), *(Z)-4-heptenal* (fatty, green odor), linalool (citrus-like, flowery), geraniol (rose-like, citrus-like), and trans-β-ionone (flowery, violet-like) have relatively high OAVs and low threshold values. Therefore, these compounds were considered to contribute more substantially to the overall odor profile [32]. Among these compounds, floral odor-active compounds such as linalool (citrus-like, flowery), (E,E)-2,4-heptadienal (fatty, flowery), benzeneacetaldehyde (fatty, flowery), and Geraniol (rose-like, citrus-like) had higher OAVs in SWT than in the others. Odor-active compounds with green odor, namely *hexanal* (green, grassy), (E)-2-nonenal (fatty, green), and cis-3-hexenyl hexanoate (fruity green odor), had higher OAVs in WWT than in the other teas.

Twenty-four odor-active compounds with FD ≥ 4 were detected. *(Z)-4-heptenal* (fatty, green odor), linalool (citrus-like, flowery), geraniol (rose-like, citrus-like), vanillin (vanilla-like, sweet), and coumarin (woodruff-like, almond paste-like) had high FD factors (i.e., FD > 128). Odor-active compounds with a floral note, such as linalool and geraniol, had high FD factors of 16–512 and 16–218, respectively. These are two key compounds producing a floral aroma [33]. A comparison of FD factors revealed that linalool and geraniol had the highest values of 512 and 256, respectively, in SWT, further explaining why the SWT had a more pronounced floral aroma. Moreover, odor-active compounds with a green odor, such as *(E)-2-hexenal* (green, apple-like), *hexanal* (green, grassy), and *(Z)-4-heptenal* (fatty, green odor), generally had higher OAVs and FD factors/odor intensity in WWT than in others, further explaining the more pronounced green characteristics of WWT aroma. Notably, some compounds with pleasant odors, such as neral (citrus, soapy), vanillin (vanilla, sweet), and coumarin (woodruff-like, almond paste-like), which were identified in WWT, SWT, and IWT by GC-O, had a lower FD factor in IWT than in the other teas. Thus, the content of these pleasant odor-active compounds that are beneficial for white tea aroma could be increased in the SWT or WWT processes. The volatile compounds in tea are produced through multiple pathways; for example, vanillin synthesis pathways include not only the shikimic acid pathway through de novo synthesis of glucose but also the oxidation of cresol, capsaicin, or vanillyl alcohol [34,35]. WWT and SWT both had additional processing compared with IWT; thus, one or more volatile synthesis pathways may have been involved, resulting in an increase in FD factors. The exact effects require further investigation.

**Figure 4:**
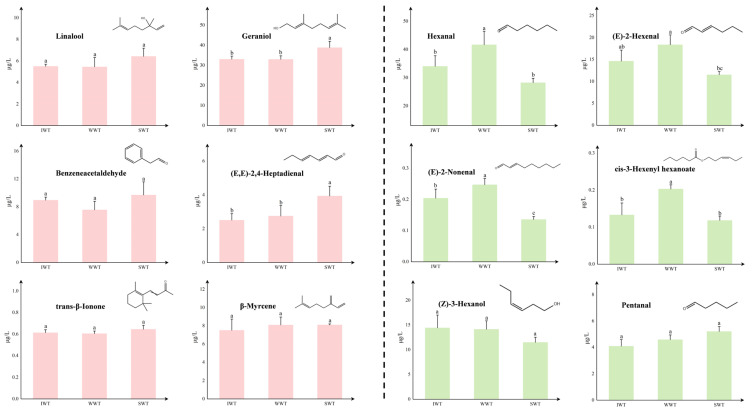
Differences in concentration of key odor-active compounds among white teas. On the left are odor-active compounds with floral odor, and on the right are odor-active compounds with green odor. Data are shown as the mean ± standard deviation (*n* = 3). Different letters on the columns represent they have significant difference (*p* < 0.05). (SWT: sunlight withering-treated white tea; WWT: withering tank withering-treated white tea; IWT: indoor withering-treated white tea).

To more visually analyze the effects of floral and green odor volatile compounds on the aroma of white tea, 12 odor-active compounds were selected for comparison of concentrations, as shown in Figure 4. The concentrations of linalool, geraniol, benzeneacetaldehyde, (E,E)-2,4-heptadienal, trans-β-Ionone, and *β-myrcene* with floral odor are shown on the left side, and it can be found that they all have higher concentrations in SWT than the other samples. The concentrations of *hexanal*, *(E)-2-hexenal*, (E)-2-nonenal, *pentanal*, *(Z)-3-hexanol*, cis-3-hexenyl hexanoate with green odor are shown on the right side, and they all have higher concentrations in the WWT than the other samples. Therefore, it can be speculated that the reason for improving the floral aroma of SWT was the increase in the concentration of odor-active compounds with floral odor caused by the sunlight withering treatment. The more pronounced grassy aroma of WWT was the increase in the concentration of odor-active compounds with grassy odor caused by the withering-tank withering treatment.

Volatile compounds with OAV ≥ 1 and FD ≥ 4 were identified as potent odorants that contribute mainly to the characteristic odor of white tea; these odor-active compounds are presented in columns 1–9 of Table 1 and are as follows: *dimethyl sulfide* (cabbage-like), *2-methyl-butanal* (malty), *1-penten-3-one* (pungent, fish), *hexanal* (green, grassy), *(Z)-4-heptenal* (fatty, green odor), *β-myrcene* (sweet balsamic aroma), linalool (citrus-like, flowery), geraniol (rose-like, citrus-like), and trans-β-ionone (flowery, violet-like). *β-Myrcene*, linalool, geraniol, and trans-β-ionone, which are key potent odorants for floral odors, exhibited higher OAVs and FD factors in SWT than in the others. However, the differences in the concentrations of *β-myrcene* and trans-β-ionone were small and insignificant, so the potent odorants that played a significant role in floral odor were linalool and geraniol. *Hexanal* is a potent odorant with green odors that exhibited high OAVs and FD factors in WWT. Therefore, we can conclude that the increased concentrations of linalool, geraniol, and *Hexanal* are the main reasons for enhancing the floral aroma of SWT and the grassy aroma of WWT. We thus tentatively speculate that the more pronounced grassy odor of WWT is due to the reduced required withering time as a result of the increased temperature during WWT; the grassy odor-active compounds in fresh leaves were not fully transformed and dissipated. Moreover, sunlight withering can significantly upregulate the expression of critical genes involved in aroma-related metabolic pathways [7]; thus, the expression of these key genes may affect the floral aroma in SWT.

### 3.4. Formation Mechanism of Key Odor-Active Compounds between Different Withering Treatments

Odor-active compounds play a key role in the overall aroma of white tea, and an understanding of their formation mechanism can improve regulation of the aroma release mechanism during white tea processing to obtain tea of higher quality. Most volatile compounds obtained in the study can be classified as fatty acid-derived volatiles (FADVs), amino acid-derived volatiles (AADVs), volatile terpenes (VTs), carotenoid-derived volatiles (CDVs), and glycosidically bound volatiles (GBVs) [14], which are produced through the shikimic acid and terpene pathways or from the oxidation of fatty acids and carotenoids.

Linalool, geraniol, and β- myrcene have a more pronounced trend as key odorants in SWT and, very interestingly, are all VTs. VTs, primarily C_10_ and C_15_ compounds, are the major contributors to the floral aroma of tea [36,37]. Isopentyl diphosphate (IPP) and its allyl isomer, dimethyl allyl diphosphate (DMAPP), are two common C_5_ terpenoid precursors [38]. These two precursors are synthesized by the cytoplasmic mevalonate (MVA) and plastid methylerythritol phosphate (MEP) pathways [39]. The key identified potent floral odorants are linalool, *β-myrcene*, and geraniol are VTs. Deng reported that the relative content of monoterpenes and sesquiterpenes in oolong tea increased significantly after SWT because of the significant upregulation of genes involved in the MEP and MVA pathways [7]. Linalool and geraniol are GBVs; Li revealed that treatment with red light withering could increase the activity of glycosidase and thus significantly improve the aroma of black tea [6]. GBV synthetases, as well as hydrolases, can strongly promote the formation of white tea aroma, and they actively participate in aroma regulation during the withering process [9]. Therefore, we speculate that the primary reason for the strong floral aroma in SWT is that sunlight exposure during withering results in an increase in VTs due to the key synthetic genes of the MEP and MVA pathways being significantly upregulated, thus enhancing the activity of related hydrolases and producing more floral odor-active compounds.

Most FADVs have a fresh or green odor [9]. The odor-active compounds *hexanal*, *pentanal*, *(Z)-3-hexanol*, (E,E)-2,4-heptadienal, and (E)-2-nonenal with green odor were among the FADVs were identified in higher concentrations in WWT. Thus, the freshness and green quality of white tea can be attributed to the increase in FADVs. Saturated and unsaturated C_6_ and C_9_ aldehydes and alcohols are also key to the production of fresh, green odor-active compounds, such as *hexanal* and *(Z)-3-hexanol* [7]. Various enzymes are involved in the production of these odor-active compounds. For example, lipoxygenase (LOX) catalyzes the production of (Z)-3-hexanal to *(E)-2-Hexenal* (leaf aldehyde) and *(Z)-3-hexanol* [40] (leaf alcohol). Hydroperoxide lyases (HPLs) can cleave hydroxides to generate C_6_ aldehydes. In potato plants, downregulation of HPL causes an increase in LOX activity and a decrease in C_6_ volatiles [41]. The more pronounced grassy note of WWT is mainly due to the increase of *hexanal* and (Z)-3-hexen-1-ol, which may be caused by the increase in AADVs and changes in enzyme activity caused by the heating and blast treatment in the withering-tank, which leads to the acceleration of the synthesis or retardation of the degradation of C_6_ or C_8_ compounds. 

Few studies to date have investigated the effects of different withering methods for white tea, and the reasons for differences in tea quality still require further, detailed research. In the future, richer and more diverse means, such as transcriptomics and proteomics, can also be used to analyze the formation mechanisms of key differential components during withering and to reveal more deeply the reasons for the aroma changes brought about by different withering methods of white tea.

## 4. Conclusions

The effects of SWT, IWT, and WWT on the aroma of white tea were investigated. The results of QDA and statistical analysis revealed significant differences in the aroma characteristics. The floral aroma was more pronounced in SWT, and the grassy flavor was more pronounced in WWT. A total of 202 volatile compounds were extracted by SAFE and HS-SPME and were subsequently analyzed with GC-MS. AEDA and OAV were used to select 35 key odor-active compounds. Nine odorants meeting FD factor ≥ 4 and OAV ≥ 1, *dimethyl sulfide*, *2-methyl-butanal*, *1-penten-3-one*, *hexanal*, *(Z)-4-heptenal*, linalool, geraniol, and trans-β-ionone, were identified as the main components affecting the aroma of white tea. By comparing the concentrations of odor-active compounds, the pronounced floral aroma in SWT can be primarily attributed to the action of geraniol, linalool, and *β-myrcene*; the pronounced grassy aroma in WWT was primarily attributed to the action of *hexanal* and *(Z)-3-hexanol*. Geraniol, linalool, and *hexanal* play a major role as potent odorants. The reasons for these differences were also investigated. SWT may result in the upregulation of key genes related to the synthesis and enhanced synthetase activity of GBVs, resulting in increased VTs content and a more pronounced floral aroma. WWT may have caused changes in the activity of enzymes that accelerate the synthesis and retard the degradation of certain compounds, resulting in an increase in FADVs. In white tea, sunlight withering can improve the floral aroma, and withering tank withering can enhance the grassy aroma. The experimental results provide a theoretical basis for selecting the withering method according to the flavor of white tea. This study reveals the effect of withering on the aroma of white tea. In the future, we can demonstrate the reasons for the differences more comprehensively by further studying the changes in key components, such as key genes and aroma precursors, during withering.

## Figures and Tables

**Table 1:** Flavor dilution factors and odor activity values for odor-active compounds in three white teas.

No.	RI ^A^	CAS	Compound ^B^	Category ^C^	Odor Characteristics	OT (μg/kg) ^D^	OAV ^E^			FD/*Odor Intensity* ^F^			OAV	FD
							IWT	WWT	SWT	IWT	WWT	SWT		
1	<600	75-18-3	*Dimethyl sulfide*	AADVs	cabbage-like	0.07	357.3	290.5	210.9	*1.8*	64/*2.7*	*2.0*	√	√
2	647	96-17-3	*2-Methyl-butanal*	AADVs	malty	1.5	5.4	4.0	3.6	*2.2*	64/*3.0*	*2.3*	√	√
3	652	1629-58-9	*1-Penten-3-one*	FADVs	pungent, fish	0.9	4.1	3.5	3.0	*2.2*	4/*2.8*	*2.2*	√	√
4	800	66-25-1	*Hexanal*	FADVs	green, grassy	1.09	31.2	38.2	25.9	*2.3*	16/*3.3*	*2.8*	√	√
5	897	6728-31-0	*(Z)-4-Heptenal*	AADVs	fatty, fish	0.0087	21.0	23.5	14.5	*2.2*	128/*3.7*	*2.5*	√	√
6	994	123-35-3	*β-Myrcene*	VTs	sweet balsamic aroma	1.2	6.3	6.7	6.7	*1.3*	8/*1.7*	*2.2*	√	√
7	1101	78-70-6	Linalool	VTs/GDVs	citrus-like, flowery	0.58	9.5	9.4	11.1	128	128	512	√	√
8	1252	106-24-1	Geraniol	VTs/GDVs	rose-like, citrus-like	1.1	30.0	30.0	35.3	16	16	256	√	√
9	1479	79-77-6	trans-β-Ionone	CDVs	flowery, violet-like	0.021	29.2	28.8	30.7	-	-	64	√	√
10	632	590-86-3	*3-Methyl-butanal*	AADVs	fruity, fatty, animal	1.44	3.1	2.6	2.2				√	
11	852	928-96-1	*(Z)-3-hexanol*	FADVs	green leaves	3.9	3.7	3.6	2.9				√	
12	928	15726-15-5	3-Methyl-4-heptanone		nutty	0.05	-	2.6	-				√	
14	1012	4313-3-5	(E,E)-2,4-Heptadienal	FADVs	fatty, flowery	0.032	78.3	85.8	122.8				√	
15	1045	122-78-1	Benzeneacetaldehyde	AADVs	fatty, flowery	5.2	1.7	1.5	1.9				√	
16	1081	586-62-9	1-Methyl-4-(1-methylethylidene)-cyclohexene		sweet-piney, oily, pleasant aroma	0.2	2.1	2.4	1.5				√	
17	1150	10340-23-5	(Z)-3-Nonen-1-ol		waxy green melon aroma	0.209	1.5	1.4	0.9				√	
18	1156	18829-56-6	(E)-2-Nonenal	FADVs	fatty, green	0.19	1.1	1.3	0.7				√	
19	1202	116-26-7	2,6,6-Trimethyl-1,3-cyclohexadiene-1-carboxaldehyde	CDVs	saffron tea	0.0455	2.2	2.7	4.6				√	
20	1377	31501-11-8	cis-3-Hexenyl hexanoate	FADVs	fruity green odour	0.195	0.7	1.0	0.6				√	
21	<600	534-22-5	*2-Methyl-Furan*		spicy smoky aroma	n.d.	-	-	-	*1.5*	8/*2.2*	*1.3*		√
22	671	110-62-3	*Pentanal*	FADVs	green, fatty, moldy	8	0.5	0.6	0.7	*1.5*	8/2.3	*1.0*		√
23	847	6728-26-3	*(E)-2-Hexenal*	FADVs	green apple-like	110	0.1	0.2	0.1	*0.8*	8/*1.3*	*1.0*		√
24	1047	695-06-7	5-Ethyldihydro-2(3H)-furanone	FADVs	coconut-like, fruity	260	0.0	0.0	0.0	4	-	8		√
25	1061	98-85-1	α-methyl-Benzenemethanol		mlid floral odor	479	-	0.0	0.0	-	2	4		√
26	1080	111-14-8	Heptanoic acid	FADVs	rancid, sweaty	640	0.0	0.0	0.0	-	-	16		√
27	1115	1960-12-8	Phenylethyl Alcohol	AADVs/GBVs	floral, honey-like	140	0.2	0.2	0.2	8	32	64		√
28	1238	106-26-3	Neral	VTs	citrus-like, soapy	5.5	0.1	0.1	0.1	8	64	32		√
29	1262	141-27-5	α-Citral	VTs	citrus-like	12	0.1	-	-	-	2	32		√
30	1365	459-80-3	Geranic acid		apple-like fruit and vegetable aroma	n.d.	-	-	-	-	4	8		√
31	1398	121-33-5	Vanillin		vanilla-like, sweet	53	0.0	0.0	0.0	16	128	128		√
32	1439	91-64-5	Coumarin	GBVs	woodruff-like, almond paste-like	11	0.2	0.2	0.2	8	4	256		√
33	1495	25524-95-2	Jasmine lactone		coconut, fatty, fruity	350	0.0	0.0	0.0	-	2	4		√
34	1521	96-76-4	2,4-Di-tert-butylphenol		phenolic-like, leather-like	500	0.0	0.0	0.0	-	-	8		√
35	1530	17092-92-1	Dihydroactindiolide	CDVs	musky or coumarin-like aroma	n.d.	-	-	-	-	2	4		√

^A^: RI, retention index. Retention indices are relative to n-alkanes on columns DB-5MS. ^B^: Odor-active compounds extracted by HS-SPME are italicized. ^C^: Odor-active compounds are classified according to their biosynthesis pathways. FADVs, fatty acid-derived volatiles; AADVs, amino acid-derived volatiles; VTs, volatile terpenes; CDVs, carotenoid-derived volatiles; GBVs, glycosidically bound volatiles. ^D^: Odor thresholds in water are in reference to the literature ([12,23,24,25). ^E^: Odor activity value, the ratio of the concentration of an odor-active compound to its odor threshold value. ^F^: Flavor dilution factor and odor intensity were evaluated by AEDA and GC–O. “-” indicates FD factor < 4 (IWT, indoor withering-treated white tea; WWT, withering-tank withering-treated white tea; SWT, sunlight withering-treated white tea; n.d., not detected).

## Data Availability

Data is contained within the article.

## Supplementary Materials

The following supporting information can be downloaded at: https://www.mdpi.com/article/10.3390/foods11162502/s1, Table S1: Moisture content of the finished withering; Table S2: Volatile compounds in the three white teas produced in this study.
