# Supplementary material for: Effects of Three Different Withering Treatments on the Aroma of White Tea
Source: Foods. 2022 Aug 19;11(16):2502. doi: 10.3390/foods11162502 (PMC9407123; doi:10.3390/foods11162502)
Supplement: Supplementary file 1 [file foods-11-02502-s001.zip › foods-1863226-supplementary.pdf]

Table S1. Moisture content of the finished withering

**Samples**[A]	**Water content (%, dw)**
SWT	10.86%±1.26%
IWT	11.97%±0.88%
WWT	11.56%±0.29%

[A]: IWT, indoor withering–treated white tea; WWT, withering-tank withering–treated white tea; SWT, sunlight withering–treated white tea.

Table S2. Volatile compounds in the three white teas produced in this study.

NO.	CAS	RI[A]	Compound	Content(μg/L) [B]						Quantitative method[C]
				IWT	SD	WWT	SD	SWT	SD	
1	75-18-3	<600	Dimethyl sulfide	25.01a	3.12	20.34b	1.84	14.76c	1.36	SPME
2	534-22-5	<600	2-Methyl-furan	0.39a	0.06	0.39a	0.04	0.29b	0.02	SPME
3	3208-16-0	<600	2-Ethyl-furan	6.7a	0.94	7.01a	0.97	4.87b	0.49	SPME
4	115-18-4	<600	2-Methyl-3-buten-2-ol	0.95a	0.23	0.96a	0.05	0.91a	0.17	SAFE
5	590-86-3	632	3-Methyl-butanal	4.43a	0.45	3.72b	0.24	3.15b	0.23	SPME
6	96-17-3	647	2-Methyl-butanal	8.09a	0.81	5.95b	0.39	5.43b	0.38	SPME
7	1629-58-9	652	1-Penten-3-one	3.85a	0.73	3.26a	0.51	2.81a	0.24	SPME
8	110-62-3	671	Pentanal	4.08bc	0.51	4.58b	0.36	5.2ab	0.38	SPME
9	616-25-1	696	1-Penten-3-ol	6.4a	0.78	4.79b	0.34	4.71b	0.67	SPME
10	513-86-0	717	Acetoin	n.d.	n.d.	n.d.	n.d.	0.24a	0.02	SAFE
11	763-32-6	735	3-Methyl-3-buten-1-ol	0.02a	0.00	0.02a	0.01	0.02a	0.00	SAFE
12	123-51-3	740	3-Methyl-1-butanol	0.93a	0.11	0.66b	0.05	0.7b	0.04	SPME
13	137-32-6	743	2-Methyl-1-butanol	1.07a	0.10	0.74b	0.05	0.73b	0.06	SPME
14	1576-87-0	759	(E)-2-Pentenal	0.79a	0.13	0.86a	0.09	0.6b	0.05	SPME
15	108-88-3	762	Toluene	10.46ab	1.70	6.8bc	1.04	8.55b	0.76	SPME
16	71-41-0	773	1-Pentanol	0.33a	0.09	0.38a	0.05	0.39a	0.02	SAFE
17	1576-95-0	776	(Z)-2-Penten-1-ol	1.16a	0.28	0.93a	0.11	1.23a	0.10	SAFE
18	66-25-1	800	Hexanal	34.02b	3.78	41.64a	4.84	28.19b	1.61	SPME
19	617-92-5	807	1-Ethyl-1H-pyrrole	0.28b	0.04	0.46a	0.06	0.2b	0.01	SPME
20	626-93-7	815	2-Hexanol	n.d.	n.d.	n.d.	n.d.	0.71a	0.02	SAFE
21	623-36-9	824	2-Methyl-2-pentenal	0.22b	0.02	0.28a	0.03	0.18b	0.01	SPME

22	4249-12-1	837	1,2,5,5-Tetramethyl-1,3-cyclopentadiene	0.03b	0.00	0.03a	0.00	0.02c	0.00	SPME
23	928-96-1	851	(Z)-3-hexanol	14.41a	2.61	14.12a	1.71	11.46a	1.07	SPME
24	100-41-4	855	Ethylbenzene	0.46a	0.07	0.46a	0.07	0.33b	0.04	SPME
25	503-74-2	856	3-Methyl-butanoic acid	n.d.	n.d.	0.2a	0.03	n.d.	n.d.	SAFE
26	505-57-7	859	2-Hexenal	n.d.	n.d.	1.88a	0.48	2.08a	0.22	SAFE
27	6728-26-3	859	(E)-2-Hexenal	14.63ab	2.53	18.36a	2.24	11.51bc	0.76	SPME
28	928-97-2	862	(E)-3-Hexen-1-ol	9.24b	0.33	8.90b	1.95	13.32a	1.57	SAFE
29	928-95-0	872	(E)-2-Hexen-1-ol	4.58a	0.89	5.17a	0.69	3.10b	0.27	SPME
30	111-27-3	876	1-Hexanol	17.97a	3.04	18.95a	2.38	12.92b	1.05	SPME
31	142-96-1	885	n-Butyl ether	1.27a	0.05	n.d.	n.d.	n.d.	n.d.	SAFE
32	110-43-0	885	2-Heptanone	0.62b	0.07	0.83a	0.11	0.54b	0.03	SPME
33	100-42-5	887	Styrene	0.95a	0.12	0.89a	0.12	0.69b	0.05	SPME
34	141-32-2	894	Butyl acrylate	0.98b	0.10	1.48a	0.23	0.77b	0.05	SPME
35	6728-31-0	897	(Z)-4-Heptenal	0.18a	0.02	0.2a	0.02	0.13b	0.01	SPME
36	111-71-7	904	Heptanal	1.68b	0.20	2.38a	0.37	1.53b	0.08	SPME
37	96-48-0	913	Butyrolactone	0.45a	0.03	0.56a	0.07	0.60a	0.04	SAFE
38	100-66-3	913	Anisole	0.14b	0.02	0.22a	0.03	0.10c	0.00	SPME
39	98-82-8	924	2-Phenyl-1-propene	n.d.	n.d.	0.10a	0.02	n.d.	n.d.	SAFE
40	106-70-7	926	Methyl hexanoate	0.17b	0.02	0.27a	0.04	0.12c	0.01	SPME
41	15726-15-5	928	3-Methyl-4-heptanone	n.d.	n.d.	0.13a	0.02	n.d.	n.d.	SAFE
42	13894-62-7	932	(Z)-3-Hexenoic acid methyl ester	n.d.	n.d.	n.d.	n.d.	0.20a	0.07	SAFE
43	587-03-1	934	3-Methylbenzyl alcohol	0.04a	0.01	0.04a	0.01	0.03a	0.00	SPME
44	928-68-7	949	6-Methyl-2-heptanone	0.24b	0.03	0.34a	0.04	0.22b	0.04	SPME
45	18829-55-5	952	(E)-2-Heptenal	0.32bc	0.05	0.41ab	0.05	0.39b	0.01	SPME

46	100-52-7	956	Benzaldehyde	2.80a	0.05	2.55a	0.25	3.16a	0.62	SAFE
47	57266-86-1	958	(Z)-2-Heptenal	n.d.	n.d.	n.d.	n.d.	0.12a	0.02	SAFE
48	111-70-6	971	1-Heptanol	0.77b	0.10	1.01a	0.12	0.63b	0.04	SPME
49	1599-47-9	976	Hexanal dimethyl acetal	6.54b	0.86	25.66a	5.89	1.06b	0.14	SAFE
50	3391-86-4	977	1-Octen-3-ol	2.73a	0.38	3.34a	0.43	3.12a	0.28	SPME
51	110-93-0	985	6-Methyl-5-hepten-2-one	0.27b	0.03	0.73a	0.06	0.67a	0.04	SAFE
52	3777-69-3	990	2-Pentyl-furan	1.59a	0.07	0.62b	0.11	1.5a	0.23	SAFE
53	123-35-3	994	β-Myrcene	10.49a	1.97	11.44a	0.92	7.25b	0.91	SPME
54	109-21-7	996	Butyl butyrate	0.48a	0.01	0.45a	0.04	n.d.	n.d.	SAFE
55	142-62-1	999	Hexanoic acid	9.37a	0.31	10.38a	2.29	9.82a	1.14	SAFE
56	124-13-0	1000	Octanal	0.64b	0.08	0.92a	0.12	0.57b	0.03	SPME
57	99-83-2	1003	α-Phellandrene	0.62b	0.11	0.7ab	0.05	0.45bc	0.08	SPME
58	4313-02-4	1008	(E,Z)-2,4-Heptadienal	1.77a	0.35	1.82a	0.23	1.26b	0.02	SPME
59	142-92-7	1010	Hecyl acetate	0.15b	0.03	0.19ab	0.02	0.14bc	0.02	SPME
60	4313-03-5	1012	(E,E)-2,4-Heptadienal	2.51b	0.38	2.75b	0.62	3.93a	0.58	SAFE
61	554-61-0	1013	2-Carene	0.41a	0.06	0.46a	0.03	0.29b	0.03	SPME
62	535-77-3	1021	1-Methyl-3-(1-methylethyl)-benzene	1.56b	0.30	2.04a	0.21	1.19b	0.15	SPME
63	138-86-3	1025	Limonene	1.63a	0.32	1.87a	0.12	1.15b	0.13	SPME
64	104-76-7	1028	2-Ethyl-1-hexanol	1.58a	0.07	n.d.	n.d.	0.79b	0.15	SAFE
65	2033-89-8	1028	3,4-Dimethoxy-phenol	0.03b	0.00	0.03a	0.00	0.04a	0.00	SPME
66	2408-37-9	1031	2,2,6-Trimethyl-cyclohexanone	0.46b	0.05	0.63a	0.08	0.46b	0.02	SPME
67	3779-61-1	1033	trans-β-Ocimene	2.46a	0.38	2.69a	0.21	1.68b	0.22	SPME
68	1669-44-9	1034	3-Octen-2-one	0.56b	0.09	0.76a	0.09	0.5b	0.03	SPME
69	100-51-6	1036	Benzyl alcohol	19.44a	1.73	20.71a	2.73	21.08a	3.47	SAFE
70	122-78-1	1045	Benzeneacetaldehyde	8.95a	0.45	7.56a	1.24	9.69a	1.89	SAFE

71	13877-91-3	1048	β-Ocimene	1.82a	0.21	2.02a	0.12	1.31b	0.13	SPME
72	695-06-7	1051	5-Ethyldihydro-2(3H)-furanone	2.36bc	0.16	2.85b	0.37	3.37ab	0.28	SAFE
73	2548-87-0	1055	(E)-2-Octenal	0.41b	0.05	0.55a	0.06	0.36b	0.02	SPME
74	98-85-1	1061	α-methyl-Benzenemethanol	n.d.	n.d.	0.79b	0.13	1.41a	0.16	SAFE
75	98-86-2	1066	Acetophenone	0.37a	0.03	0.19b	0.01	0.15c	0.00	SPME
76	1000373-80-3	1068	Ethyl 2-(5-methyl-5-vinyltetrahydrofuran-2-yl)propan-2-yl carbonate	6.94ab	0.75	6.84b	1.04	5.31bc	0.46	SPME
77	111-87-5	1069	1-Octanol	1.14b	0.18	1.45ab	0.18	0.96bc	0.12	SPME
78	30086-02-3	1069	(E,E)-3,5-Octadien-2-one	0.77ab	0.18	0.58b	0.13	0.45bc	0.03	SAFE
79	5989-33-3	1072	cis-Linalool oxide	5.13a	0.23	4.94a	0.96	1.08b	0.13	SAFE
80	10032-05-0	1075	1,1-Dimethoxy-heptane	n.d.	n.d.	n.d.	n.d.	1.26a	0.24	SAFE
81	111-14-8	1080	Heptanoic acid	0.53a	0.13	0.77a	0.03	0.81a	0.23	SAFE
82	586-62-9	1081	1-Methyl-4-(1-methylethylidene)-cyclohexene	0.43a	0.06	0.48a	0.03	0.30b	0.04	SPME
83	1195-32-0	1087	1-Methyl-4-(1-methylethenyl)-benzene	0.27b	0.03	0.38a	0.03	0.23b	0.02	SPME
84	34995-77-2	1089	trans-Linalool oxide (furanoid)	10.51a	0.73	10.4a	1.58	10.92a	1.46	SAFE
85	38284-27-4	1094	3,5-Octadien-2-one	1.45c	0.26	1.97b	0.49	2.45ab	0.21	SAFE
86	93-58-3	1097	Methyl benzoate	n.d.	n.d.	n.d.	n.d.	0.12a	0.00	SAFE
87	78-70-6	1101	Linalool	5.5a	0.20	5.45a	0.87	6.42a	0.77	SAFE
88	124-19-6	1104	Nonanal	2.56a	0.25	2.77a	0.31	1.87b	0.08	SPME
89	29957-43-5	1104	3,7-Dimethyl-1,5,7-octatrien-3-ol	1.25a	0.22	0.25b	0.01	n.d.	n.d.	SAFE
90	5337-72-4	1112	2,6-Dimethyl-cyclohexanol	1.29a	0.11	1.52a	0.17	1.35a	0.21	SAFE
91	60-12-8	1115	Phenylethyl Alcohol	26.42a	1.24	27.87a	2.20	28.65a	3.89	SAFE

92	432-24-6	1115	2,6,6-Trimethyl-2-cyclohexene-1-carboxaldehyde	0.04b	0.01	0.05ab	0.01	0.04bc	0.00	SPME
93	111-11-5	1122	Caprylic acid methyl ester	n.d.	n.d.	n.d.	n.d.	0.16a	0.05	SAFE
94	7216-56-0	1126	(4E,6Z)-2,6-Dimethyl-2,4,6-octatriene	0.8a	0.15	0.95a	0.08	0.58b	0.07	SPME
95	460-01-5	1127	(3E,5E)-2,6-Dimethyl-1,3,5,7-octatetrene	0.26a	0.03	0.32a	0.03	0.2b	0.03	SPME
96	140-29-4	1139	Benzyl nitrile	0.08b	0.02	0.12a	0.02	0.07b	0.01	SAFE
97	1125-21-9	1145	2,6,6-Trimethyl-2-cyclohexene-1,4-dione	0.07a	0.00	n.d.	n.d.	0.07a	0.01	SAFE
98	10340-23-5	1150	(Z)-3-Nonen-1-ol	0.32a	0.05	0.29a	0.04	0.19b	0.03	SPME
99	18829-56-6	1156	(E)-2-Nonenal	0.2b	0.03	0.25a	0.02	0.14c	0.01	SPME
100	1000414-18-0	1158	Isoneral	0.09b	0.01	0.11a	0.02	0.06c	0.01	SPME
101	143-08-8	1169	1-Nonanol	0.80b	0.16	0.95ab	0.10	0.65bc	0.04	SPME
102	14009-71-3	1172	(3R,6R)-2,2,6-Trimethyl-6-vinyltetrahydro-2H-pyran-3-ol	n.d.	n.d.	n.d.	n.d.	3.04a	0.47	SAFE
103	39028-58-5	1172	(3R,6S)-2,2,6-Trimethyl-6-vinyltetrahydro-2H-pyran-3-ol	7.23a	0.26	2.44b	0.33	7.84a	1.28	SAFE
104	124-07-2	1174	Octanoic acid	1.00a	0.14	0.91a	0.24	1.12a	0.18	SAFE
105	14049-11-7	1177	2H-Pyran-3-ol, 6-ethenyltetrahydro-2,2,6-trimethyl-	7.2a	0.31	7.22a	0.58	n.d.	n.d.	SAFE
106	55722-59-3	1177	3,7-Dimethyl-3,6-octadienal	0.17a	0.03	0.21a	0.02	0.12b	0.01	SPME
107	91-20-3	1178	Naphthalene	0.42a	0.04	0.45a	0.01	0.29b	0.06	SPME
108	53398-84-8	1183	(E)-hex-3-enyl butyrate	0.41a	0.06	0.42a	0.05	0.23b	0.01	SPME
109	585-74-0	1188	1-(3-methylphenyl)-Ethanone	0.03a	0.00	n.d.	n.d.	n.d.	n.d.	SAFE
110	98-55-5	1191	α-Terpineol	0.26a	0.05	0.26a	0.02	0.18v	0.01	SPME
111	119-36-8	1194	Methyl salicylate	7.73a	0.15	7.84a	1.10	8.92a	1.48	SAFE

112	112-40-3	1199	Dodecane	0.07a	0.01	0.09a	0.02	0.04b	0.00	SPME
113	499-74-1	1201	Carvenone	0.08ab	0.01	0.05bc	0.01	0.06b	0.01	SPME
114	116-26-7	1202	2,6,6-Trimethyl-1,3-cyclohexadiene-1-carboxaldehyde	0.10b	0.02	0.12b	0.02	0.21a	0.05	SAFE
115	74410-00-7	1203	trans-isopiperitenol	0.26a	0.07	0.22a	0.06	n.d.	n.d.	SAFE
116	112-31-2	1207	Decanal	0.73a	0.10	0.47b	0.06	0.54b	0.06	SPME
117	1000190-36-8	1208	5-Isopropenyl-2-methylcyclopent-1-enecarboxaldehyde	0.05b	0.01	0.07a	0.01	0.04b	0.01	SPME
118	101-48-4	1217	(2,2-Dimethoxyethyl)-benzene	0.71b	0.13	2.45a	0.22	0.12c	0.02	SAFE
119	122-99-6	1221	2-Phenoxy-ethanol	n.d.	n.d.	n.d.	n.d.	1.02a	0.30	SAFE
120	432-25-7	1222	2,6,6-Trimethyl-1-cyclohexene-1-carboxaldehyde	0.12a	0.00	0.12a	0.01	0.12a	0.02	SAFE
121	1731-84-6	1222	Methyl nonanoate	0.05b	0.01	n.d.	n.d.	0.25a	0.07	SAFE
122	106-25-2	1225	Nerol	0.76a	0.04	0.83a	0.10	0.88a	0.13	SAFE
123	53398-85-9	1227	cis-3-Hexenyl-α-methylbutyrate	0.33b	0.05	0.59a	0.06	0.32b	0.01	SPME
124	35154-45-1	1232	cis-3-Hexenyl isovalerate	1.92a	0.22	1.84a	0.17	1.36b	0.07	SPME
125	20189-42-8	1233	3-Ethyl-4-methyl-1H-pyrrole-2,5-dione	0.72a	0.04	0.68a	0.12	0.89a	0.15	SAFE
126	106-26-3	1238	Neral	0.56a	0.14	0.49a	0.08	0.53a	0.05	SAFE
127	106-24-1	1252	Geraniol	37.97a	3.92	35.55a	2.30	34.7a	5.83	SAFE
128	3913-81-3	1259	(E)-2-Decenal	0.26b	0.04	0.32ab	0.03	0.23bc	0.01	SPME
129	21494-57-5	1260	3-Ethenyl-4-methyl-1H-Pyrrole-2,5-dione	0.67a	0.07	0.60a	0.01	0.69a	0.07	SAFE
130	105-60-2	1261	Caprolactam	0.71a	0.10	n.d.	n.d.	n.d.	n.d.	SAFE
131	141-27-5	1267	α-Citral	1.12a	0.34	n.d.	n.d.	n.d.	n.d.	SAFE
132	5392-40-5	1268	Citral	8.91b	1.65	10.72ab	1.52	6.28bc	0.93	SPME

133	112-05-0	1270	Nonanoic acid	1.19a	0.13	1.14a	0.08	1.24a	0.24	SAFE
134	89-83-8	1290	Thymol	n.d.	n.d.	n.d.	n.d.	0.05a	0.01	SAFE
135	536-60-7	1293	p-Cymen-7-ol	0.08ab	0.01	n.d.	n.d.	0.08a	0.01	SAFE
136	105-86-2	1297	Geranyl formate	n.d.	n.d.	n.d.	n.d.	0.12a	0.01	SAFE
137	629-50-5	1298	Tridecane	0.04a	0.01	0.05ab	0.01	0.03a	0.00	SPME
138	761-65-9	1301	N,N-dibutyl-formamide	0.18a	0.04	n.d.	n.d.	0.16a	0.03	SAFE
139	7786-61-0	1313	2-Methoxy-4-vinylphenol	n.d.	n.d.	1.45a	0.41	n.d.	n.d.	SAFE
140	55955-46-9	1314	4-(2,6,6-Trimethyl-2-hydroxycyclohexyl)-3-buten-2-one	0.17a	0.03	0.18a	0.02	0.14a	0.02	SPME
141	1189-09-9	1320	trans-Geranic acid methyl ester	1.74b	0.21	2.36a	0.23	1.39b	0.09	SPME
142	606-45-1	1333	Methyl 2-methoxybenzoate	0.06b	0.00	0.09b	0.02	0.13a	0.02	SAFE
143	1000154-64-0	1337	5,6-Diethyl-cyclohexa-1,3-diene	0.14b	0.02	0.17a	0.02	0.11b	0.01	SPME
144	673-84-7	1341	2,6-Dimethyl-2,4,6-octatriene	0.03c	0.00	0.08a	0.01	0.05b	0.00	SPME
145	64142-78-5	1343	2,6-Dimethyl-2,7-octadiene-1,6-diol	0.42a	0.02	n.d.	n.d.	n.d.	n.d.	SAFE
146	97-53-0	1352	Eugenol	0.06a	0.01	n.d.	n.d.	n.d.	n.d.	SAFE
147	30364-38-6	1356	1,2-Dihydro-1,1,6-trimethyl-naphthalene	n.d.	n.d.	n.d.	n.d.	0.04a	0.01	SAFE
148	104-61-0	1360	γ-lactone	0.5a	0.06	0.46	0.04	n.d.	n.d.	SAFE
149	459-80-3	1365	Geranic acid	39.86a	0.95	38.81a	4.00	39.46a	8.47	SAFE
150	334-48-5	1371	n-Decanoic acid	0.22a	0.04	0.2a	0.02	0.22a	0.02	SAFE
151	105-87-3	1375	Geranyl acetate	0.15b	0.01	0.22a	0.03	0.13b	0.01	SPME
152	31501-11-8	1377	cis-3-Hexenyl hexanoate	0.13b	0.03	0.20a	0.01	0.12b	0.01	SPME
153	488-10-8	1395	Jasmone	0.32bc	0.05	0.45a	0.05	0.26b	0.04	SPME

154	121-33-5	1398	Vanillin	1.48b	0.04	1.31b	0.15	1.90a	0.29	SAFE
155	575-41-7	1402	1,3-Dimethyl-naphthalene	0.04a	0.00	n.d.	n.d.	n.d.	n.d.	SAFE
156	7768-28-7	1411	2-Hydroxy-benzeneethanol	0.05a	0.00	n.d.	n.d.	n.d.	n.d.	SAFE
157	35692-94-5	1422	4-Hydroxy-2,6,6-trimethylcyclohex-1-enecarbaldehyde	0.16a	0.01	n.d.	n.d.	n.d.	n.d.	SAFE
158	127-41-3	1427	α-Ionone	0.25b	0.04	0.34a	0.05	0.18b	0.02	SPME
159	91-64-5	1439	Coumarin	2.09a	0.10	2.04a	0.21	2.55a	0.39	SAFE
160	689-67-8	1447	6,10-Dimethyl-5,9-undecadien-2-one	n.d.	n.d.	n.d.	n.d.	0.19a	0.00	SAFE
161	3796-70-1	1447	Geranylacetone	1.42a	0.17	1.71a	0.29	0.59b	0.03	SPME
162	131-11-3	1448	Dimethyl phthalate	0.31b	0.03	0.27bc	0.05	0.4ab	0.06	SAFE
163	719-22-2	1461	2,6-Di-tert-butyl-p-benzoquinone	n.d.	n.d.	n.d.	n.d.	0.06a	0.01	SAFE
164	10396-80-2	1476	2,6-Di-tert-butyl-4-hydroxy-4-methylcyclohexa-2,5-dien-1-one	0.08b	0.02	0.11a	0.01	0.06b	0.01	SPME
165	79-77-6	1479	trans-β-Ionone	0.70a	0.06	0.68a	0.07	0.57a	0.13	SAFE
166	62108-16-1	1481	2,3-Dihydro-4-methyl-1H-indole	2.36a	0.09	n.d.	n.d.	n.d.	n.d.	SAFE
167	23267-57-4	1482	β-Ionone epoxide	2.31a	0.11	2.58a	0.41	2.22a	0.45	SAFE
168	25524-95-2	1489	Jasmine lactone	1.13a	0.08	0.67b	0.14	0.62b	0.08	SAFE
169	96-76-4	1505	2,4-Di-tert-butylphenol	8.24a	0.89	2.22b	0.43	11.62a	2.84	SAFE
170	111-82-0	1523	Methyl dodecanoate	0.03b	0.00	n.d.	n.d.	0.08a	0.02	SAFE
171	17092-92-1	1530	Dihydroactindiolide	3.32a	0.06	3.37a	0.53	3.76a	0.68	SAFE
172	5471-51-2	1553	4-(4-hydroxyphenyl)-2-butanone	n.d.	n.d.	0.57a	0.03	n.d.	n.d.	SAFE
173	143-07-7	1561	Dodecanoic acid	n.d.	n.d.	0.22a	0.02	0.28a	0.06	SAFE
174	40716-66-3	1565	Nerolidol	0.13b	0.02	0.21a	0.01	0.11b	0.02	SPME

175	25152-85-6	1575	cis-3-Hexenyl benzoate	0.12a	0.02	0.11a	0.00	0.08b	0.00	SPME
176	84-66-2	1586	Diethyl Phthalate	n.d.	n.d.	0.14a	0.04	0.16a	0.01	SAFE
177	6846-50-0	1586	2,2,4-Trimethyl-1,3-pentanediol diisobutyrate	0.17b	0.03	0.29a	0.06	0.12b	0.03	SAFE
178	1211-29-6	1642	Methyl jasmonate	0.08a	0.01	0.07a	0.00	n.d.	n.d.	SAFE
179	2305-13-7	1644	4-Hydroxy-3-methoxy-benzenepropanol	1.40ab	0.16	1.11b	0.33	0.87bc	0.07	SAFE
180	77-93-0	1653	Triethyl citrate	0.09a	0.01	0.04b	0.01	n.d.	n.d.	SAFE
181	27185-77-9	1658	3-Keto-β-ionone	n.d.	n.d.	0.09a	0.01	n.d.	n.d.	SAFE
182	38274-01-0	1684	3-Hydroxy-5,6-epoxy-β-ionone	6.41a	0.61	4.2b	0.27	4.56b	0.75	SAFE
183	20675-95-0	1693	(E)-2,6-Dimethoxy-4-(prop-1-en-1-yl)phenol	0.12b	0.00	n.d.	n.d.	0.59a	0.07	SAFE
184	531-59-9	1726	7-Methoxy-2H-1-benzopyran-2-one	3.92a	0.37	2.63b	0.15	2.65b	0.38	SAFE
185	127321-19-1	1733	4-Hydroxy-2-methoxycinnamaldehyde	0.39a	0.08	0.32a	0.05	n.d.	-	SAFE
186	1620-98-0	1755	3,5-di-tert-Butyl-4-hydroxybenzaldehyde	0.19b	0.02	0.05c	0.01	0.27a	0.06	SAFE
187	73410-02-3	1768	6-Hydroxy-4,4,7a-trimethyl-5,6,7,7a-tetrahydrobenzofuran-2(4H)-one	3.14a	0.21	2.88a	0.34	1.93b	0.33	SAFE
188	110-27-0	1824	Isopropyl myristate	0.1a	0.02	0.04b	0.01	n.d.	-	SAFE
189	84-69-5	1861	Diisobutyl phthalate	n.d.	n.d.	n.d.	n.d.	2.32a	0.20	SAFE
190	82304-66-3	1909	7,9-Di-tert-butyl-1-oxaspiro(4,5)deca-6,9-diene-2,8-dione	0.26a	0.01	0.10b	0.02	0.26a	0.08	SAFE
191	112-39-0	1935	Methyl palmitate	0.05a	0.01	0.05a	0.01	0.06a	0.01	SAFE
192	6386-38-5	1941	Methyl 3-(3,5-di-tert-butyl-4-hydroxyphenyl)propionate	n.d.	n.d.	0.01a	0.00	0.01a	0.00	SAFE

193	84-74-2	1969	Dibutyl phthalate	2.7b	0.77	2.38b	0.11	7.58a	1.66	SAFE
194	57-10-3	1981	n-Hexadecanoic acid	0.06b	0.03	n.d.	n.d.	0.12a	0.03	SAFE
195	142-91-6	2029	Isopropyl palmitate	0.03a	0.01	n.d.	n.d.	n.d.	n.d.	SAFE
196	112-61-8	2126	Methyl stearate	0.14bc	0.03	0.18b	0.02	0.22ab	0.05	SAFE
197	629-54-9	2181	Hexadecanamide	0.57a	0.14	n.d.	n.d.	0.41a	0.07	SAFE
198	77-90-7	2244	Tributyl acetylcitrate	0.10a	0.02	0.03b	0.00	n.d.	n.d.	SAFE
199	83834-59-7	2319	2-Ethylhexyl trans-4-methoxycinnamate	0.48a	0.04	0.31b	0.08	0.38b	0.07	SAFE
200	301-02-0	2360	(Z)-9-Octadecenamide	1.04a	0.22	0.35b	0.01	0.61b	0.14	SAFE
201	124-26-5	2388	Octadecanamide	n.d.	n.d.	n.d.	n.d.	0.32a	0.08	SAFE
202	1000377-93-5	2527	Phthalic acid, di(2-propylpentyl) ester	1.56ab	0.47	0.86bc	0.10	1.19b	0.08	SAFE

A: RI, retention index. Retention indices relative to n-alkanes on columns DB-5MS.

B: IWT, indoor withering–treated white tea; WWT, withering-tank withering–treated white tea; SWT, sunlight withering–treated white tea. Standard deviation of concentrations of volatile compounds. (n = 3), SD, standard deviation; n.d., not detected in sample. Values with different superscript roman letters (a, b, c) in the same row are significantly different according to the Duncan test ($p \leq 0.05$).

C: SPME, volatile compounds quantified by headspace solid-phase microextraction; SAFE, volatile compounds quantified by solvent-assisted flavor evaporation.
